# Falling upward with Parkinson’s disease

**DOI:** 10.1038/s41531-017-0031-3

**Published:** 2017-09-13

**Authors:** Stephen A. Buetow, Pablo Martínez-Martín, Brendan McCormack

**Affiliations:** 1 Department of General Practice and Primary Health Care, University of Auckland, Auckland, New Zealand; 2 National Center of Epidemiology and Centro Investigación Biomédica en Red Enfermedades Neurodegenerativas (CINERNED), Madrid, Spain; 3 Division of Nursing, and Centre for Person-centred Practice Research, School of Health Sciences, Queen Margaret University, Musselburgh, EH21 6UU UK

## Abstract

Falls can injure, even kill. No one with Parkinson’s disease (PD) wants to fall by accident. However, the potential nastiness of falls does not preclude a more nuanced understanding of the personal meaning that falls can have. Rather than view falls as a problem to fear and manage solely by preventing and repairing harm, people with PD and those who care for them may recast falls as a mixed blessing. Falls may be a resource, skill, and catalyst for personal growth. We discuss how falls may give rise to opportunities in interrelated domains: capabilities, credo, character, creativity, chronemics, and connectedness. Clinicians could incorporate a positive focus across these domains to help people with PD to ‘fall upward’ in the sense of flourish.

## Introduction

Does focusing on falls as a problem without redeeming features do people who fall a disservice? Provocatively, we answer ‘yes’. Although no one wants to fall by accident, the potential nastiness of falls does not preclude them from also being a resource and catalyst for growth by those who experience them. We seek to defend this even-handed perspective, specifically for people with Parkinson’s disease (PD).

Accidental falls are a key indicator of injury and disability in people with PD.^1^ Clinicians seek to limit this harm. Their focus is on falls prevention, recovery of patient function, and minimizing disability and dependence. Put simply, therefore, medical models of disability view falls as a problem to fix and try to avoid.^2^ Without diminishing the serious harm that falls with PD commonly cause, this paper disavows a fully disabling meaning of falls. It complements a deficit focus on falls with a strengths perspective on how falls may stimulate people with PD to review their life situation and set challenging but achievable goals. These goals include flourishing or reaching for the highest good in terms of personal capabilities.

Disciplines including theology, philosophy, and psychology have long recognized that some people flourish from personal experience of adversity. The possibility of flourishing through falls, not just despite them, is especially relevant to PD. Slow progress has been made in preventing falls in people with this condition, who may perceive falls as one of the worst aspects of having PD.^3^ Dopaminergic medications—the mainstay of current drug treatment for PD—are ineffective at improving balance and reducing falls.^4^ Rehabilitative approaches may improve balance in PD, but, despite exceptions,^5, 6^ without reducing falls.^7, 8^ Therefore, many people with PD experience recurrent falls.^9^ Fear of falling exacerbates this problem,^10^ even though this fear is also associated with restricting or avoiding participating in daily activities that are important for independence, quality of life, and lengthened survival.^11^

An opportunity exists to delineate domains in which falls and their management may benefit people with PD. Consistent with the development of positive neurology,^12^ this perspective indicates potential opportunities in interrelated domains: capabilities, credo, character, creativity, chronemics, and connectedness. Clinicians could incorporate a positive focus across these domains to assist people with PD to ‘fall upward’ in the sense of flourishing. Beyond offering guidance and motivation, this person-centered ideal could inspire people living with PD and those caring for them.

## Capabilities

Sick role theory^13^ suggests that sick people can benefit from society not expecting them to fulfill their normal social roles. Therefore, falls with PD may restrict what society expects of people with PD. However, falls may also prick the pride of people with PD to challenge this social expectation. Focusing on what they can do rather than what they can’t, they can set goals that are inherently rewarding to optimize their personal capabilities. For example, a corollary of ‘Parkinson’s law’ (as propounded by C. Northcote Parkinson, not James Parkinson who established as recognizable the condition renamed Parkinson’s disease) is that setting ambitious but achievable goals^14^ can motivate people to close the gap between what they are currently able to do and what they want.^15^ This motivation resonates with goal-setting theory,^14^ with capability theory that focuses on people realizing their capabilities to be and do what they have reason to value,^16, 17^ and with a strengths model that focuses on disability as a resource in the face of adversity.^18^ People who fall with PD, for example, may use external cues to compensate for deficits in their ambulation and act on goals relating to movement in order to enhance their well-being.

The cues, independent of whether they are neuroprotective and decrease falls, can enable people, who might otherwise struggle to initiate and perform movements like walking, to perform activities even more complex and motivating. For example, these people may catch and throw, cycle^19, 20^ and even run up stairs.^21^ Potentially moving beyond baseline levels of functioning and activity, they may take up activities such as dance,^22^ become physically active,^8^ increase their fitness and add meaning to their lives and identity. They may augment this meaning by using physical activity to improve their non-motor symptoms^23^ and adopt a proportionate attitude to falls.

## Credo

Among people with PD, subjective well-being and a composed attitude may palliate physical discomfort from falling, improve executive control of balance systems and gait,^24^ and provide a cognitive resource for uptake of, and adherence to, falls-related interventions. Cross-sectional data^25^ for community dwelling older people, although not with PD, indicate that with composure, appropriate fear of falling can also be a benefit of falls. The fear reduces future falls risk by helping people, who commonly misjudge their actual gait ability at older ages,^26^ not to overestimate their falls efficacy (ability to maintain their balance) amid their physical limitations. From this perspective, ‘falling is not an error, it is a skill’^27^ that experience of falling can develop to release the intrinsic capability of people to make change with which they feel at ease. As a challenge of adaptive capacity, falls may motivate people with PD to revise care plans, including their medication regimen and home setup, and optimize quality of life.^28^ Such opportunities for growth motivate people to move on by setting challenging goals that push them to their limits in a good way^14^ and strengthen them as people. A perhaps surprising resource in this prescription is uncertainty.

Incomplete and subjective information about the circumstances of a fall commonly informs its clinical management.^29^ This uncertainty can provide hope, facilitate faith, and make subjectively rational the experience and appreciation of positive emotions such as joy. In turn, disciplines spanning psychology, biology, and neuroscience^30^ show that people’s expectations can alter their reporting of embodied experience, for example, of pain from injury through falls.^31^ Despite hurting, pain can usefully signal to people a need to protect body parts that the brain thinks are damaged.^32^ Apart from suffering, which ‘can be constructive, perhaps even redemptive’,^33^ pain appears to help some people to feel alive when they are emotionally numb,^34^ and enjoy physical and emotional relief from endorphin release in response to pain. Yet, pain can vary in its meaning to, and tolerance by, people.

## Character

Positive attitudes are conducive to people cultivating and exercising personal virtues as stable traits of good character. People with PD may find that falling is humbling and increases the compassion that they and others feel. They may stoically endure discomfort and continue, with appropriate modifications and support, to engage bravely in meaningful activities. It is because falls can be terrible that they may also remind people to count their blessings. Without falling, people might not be alerted to a change in their health condition; receive care such as a risk assessment for falling; adopt interventions, such as a hip protector to promote bone health and reduce the risk of fall-related injury; be prompted to make the most of their life; and enjoy praise for their fortitude. Informing these virtues is the Japanese art of *ukemi*, falling safely.

Practitioners of martial arts, such as Judo and Aikido, study *ukemi* to be prepared to fall but also to cease resisting falls. For judo exponents, for example, a fall may indicate not defeat but rather, *sutemi* or sacrifice of their standing position in order to defeat an adversary. Similarly, people with PD may sacrifice fear of falling by learning to ‘throw away’ the self. This retreat of ego helps them to relax their body, stay as alert as possible to experience of a fall^35^ and fall safely in this mindful state.^36^

## Creativity

PD is sometimes associated with a distinctive but disputed personality type^37^ that coincides with, or expresses itself before, a diagnosis of PD. Key traits of this personality profile include weak novelty seeking amid precautionary behavior. These traits may help to account for the paradox that, in producing motor and cognitive deficits, PD could predispose some people to interpret falls prudently and improvize. Other explanations for their creativity include sublimation and disinhibition,^38^ as well as stimulation from treatment for PD. Compared to not living with PD, levodopa, and dopamine agonists,^39^ as well as lifestyle activities such as dance,^27^ may release and exercise preserved creative potential for goal setting. Independent of impulse control disorders, including dopamine-dysregulation syndrome,^40^ the boost to creativity confers potential benefits. It may enable people with PD to improve control over, and coordinate their movement; use continuous adaptive movement to recover intermittent and unexpected losses of balance; confidently manage postural instability;^27^ enhance quality of life and support successful aging, including the treatment of comorbid states such as depression.^41^

## Chronemics

PD slows everything down. Falls accentuate this effect when they restrict physical movement. It is common to view this slowness or absence of movement as a problem. Things take longer to complete, which can be frustrating and increase inactivity. However, slowing down through PD and falls could also produce benefits. In a busy world that moves increasingly fast, slowing down could enable people with PD to become more present to, and aware of, their environment and themselves. No-one says it better than the poet, William Henry Davies: ‘What is this life, full of care, we have no time to stand and stare’. Falls with PD could offer people this time to notice the world and reflect on what is important to them. Realizing that falls do not define their identity—and constructing activities, such as reading, as therapeutic—they may feel uplifted, relax, and counteract distress. These benefits are health promoting and may even function to reduce falls and PD symptoms such as tremors.^42^ With relaxation^43^ and mindfulness,^44^ they may also reduce a need for sedatives that increase balance problems and tiredness, and report PD-associated improvements in daily activity and quality of life—although the effectiveness of these interventions is still uncertain. Contrariwise, people with PD could make time appear to quicken. They may cease to experience time as moving slowly by becoming less conscious of themselves,^45^ for example through full immersion in what they do.^46^

## Connectedness

Beyond the need sometimes to disconnect from daily life is the power of falls to facilitate meaningful connections, including social and spiritual relationships. As stressful events, falls may bring people with PD closer to others and meet a fundamental human need for interaction and connection,^47^ via shared experience and empathy. This psychosocial relatedness and bonding may support those who fall, directly through receiving care for falls but also indirectly through the opportunity to reciprocate or offer care to others. Despite a lack of evidence that social support prevents falls,^48^ people who fall may express altruism via gratitude, participating in research, and teaching others how to minimize falls risk and fall safely. In turn the enhanced well-being of these others is morally important for itself and for developing their capacity to support the welfare and identity of the person who has fallen.^49^

## Implications

A clinical implication is the need to broaden care of PD (and other medical conditions) beyond remediation of totally negative meanings of falls and other manifestations of PD. Reaching beyond a biomedical and reductionist understanding of falls, this clinical care could accommodate nuanced meanings of falls. Rather than view falls as a form of failure to fear and manage solely by preventing and repairing harm, clinical practice could recast them as a mixed blessing. Without discounting the harm that can result from falls, people with PD could use them to take stock of their life and changing identity. In turn, clinicians could support these people, for example by informing their attitude to falls; helping them discover their inner resources and strengths, including those associated with PD; use their capabilities to help compensate for, and grow from, falls; inform inclusive and superior design options for others at risk of falls; recall and share successes, for example within support groups; and focus on what works, not just what does not. This multifaceted approach could move people falling with conditions like PD beyond recovery and rehabilitation toward flourishing—paradoxically because PD and falls cause distress.
